# Structural Characterization of Protein–Nucleic Acid Complexes: An Overview of the Recent Innovation in the Analytic Methods

**DOI:** 10.3390/ijms262311465

**Published:** 2025-11-26

**Authors:** Maria Laura Bellone, Francesca Mensitieri, Elvira Marmo, Alessia Nunzia Calabrese, Giulia Gaudino, Viviana Izzo, Fabrizio Dal Piaz

**Affiliations:** 1 Department of Pharmacy, University of Salerno, Via Giovanni Paolo II, 84084 Fisciano, Italy; 2 Department of Medicine and Surgery, University of Salerno, Via Giovanni Paolo II, 84081 Baronissi, Italy; emarmo@unisa.it (E.M.); alcalabrese@unisa.it (A.N.C.); ggaudino@unisa.it (G.G.); vizzo@unisa.it (V.I.); fdalpiaz@unisa.it (F.D.P.); 3 School of Specialization in Clinical Pathology and Clinical Biochemistry, University of Salerno, Via Giovanni Paolo II, 84081 Baronissi, Italy; 4 AOU San Giovanni di Dio e Ruggi d’Aragona Hospital, 84131 Salerno, Italy

**Keywords:** microscopy, cryo-EM, coimmunoprecipitation, proximity label, surface plasmon resonance

## Abstract

The study of gene expression regulation systems, transcriptional, post-transcriptional, and translational processes require in-depth knowledge of the structure and dynamics of protein–DNA and protein–RNA complexes. Furthermore, the discovery of the multiple roles played by different types of RNA, including within extracellular vesicles, has raised new questions about the systems responsible for stabilizing and transporting these RNAs. Over the years, numerous experimental approaches have been developed for the study of complexes between proteins and nucleic acids, both in terms of the type and degree of accuracy of the information they are able to provide. Furthermore, some techniques have proven suitable for monitoring dynamic processes, while others provide very high-resolution data. Finally, the different methods also differ in their applicability directly to the study of complexes within their biological environment, while others can only be used on purified samples. The purpose of this review is to provide an overview of many of these approaches, accompanied by some examples of recent applications, to highlight their strengths and limitations.

## 1. Introduction

The central dogma of molecular biology is that “DNA makes RNA and RNA makes proteins” as a single stream of genetic information. Over time, this statement has been dismissed as rather simplistic and far from reality. Deep understanding of cellular processes has led to the idea that cellular functions are maintained by dynamic interactions between all macromolecules at different levels [1]. In particular, the dynamic interactions of multiprotein complexes with DNA or RNA nucleic acids have been defined as key players in the cellular context [2]. Based on the importance of protein–nucleic acid interactions in all aspects of the functioning of biological systems grows, the amount of crucial information still missing becomes increasingly evident. Describing a protein–nucleic acid complex implies to provide information—possibly time resolved—ranging from the identity of the molecules involved (which may be as few as two, but also dozens or more), to the definition of the protein domains in direct contact with the nucleic acid and the nucleic acid motifs recognized by the proteins, all the way to the conformational rearrangements that occur in proteins and the structure of nucleic acids.

The key role played by proteins in chromatin structure has been postulated at the beginning of the last century, and the hypothesis that histones were involved in DNA biological activity was formulated in 1950 [3]. Protein binding to DNA regulates different aspects of gene expression, duplication, and repair [4], being crucial for both physiological and pathological processes. Several DNA-binding proteins have been described, including helicases, transcriptional factors, polymerases, kinases, and phosphatases, but only for some of these have the molecular mechanisms underlying their interaction with nucleic acids been elucidated [5]. Even more complicated, the investigation of the vast galaxy of RNA–protein interactions appear. In fact, in addition to the ever-increasing number of RNA-binding proteins with different roles that are being discovered almost daily, there is also the structural, functional, and localization heterogeneity of RNAs. RNA–protein interactions represent critical nodes in a complex regulatory network where they are fundamental to various cellular processes, including RNA processing, RNA transport, gene expression regulation, translation, as well as RNA transport and RNA decay [6]. Ribonucleoprotein complexes, indeed, include large cytosolic ribosomes as well as small mRNAs interacting with an RNA-binding protein within exosomes [7,8].

In this context, it is not surprising that there is an incessant search for new experimental approaches that allow us to obtain increasingly accurate and reliable data on protein–RNA and protein–DNA interactions. A wide range of well-defined techniques and approaches are available, each with their own strengths and limitations. The aim of this review is to provide a critical overview of the most efficient experimental procedures for exploring protein–nucleic acid interactions in depth, with a particular focus on the structural aspects of the resulting complexes.

## 2. Microscopy-Based Methods

Most biological processes are characterized by widely different biomolecular interactions that take part in numerous essential molecular events, such as protein–protein interactions or protein–nucleic acid interactions. Given their biological critical role, many studies aim to develop increasingly precise and accurate analytical techniques to improve their detection and to better understand their behavior.

Traditional biochemical assays do not reveal how DNA changes shape when a protein binds a specific oligonucleotide sequence. The way a protein binds to and distorts the shape of DNA is crucial to its function. Microscopy techniques can fill this gap by providing a precise and comprehensive image of the protein–DNA complexes structure and dynamics. One of the most important advantages of these techniques, which include force-based methodologies (such as atomic force microscopy, AFM) and fluorescence-based methods (such as single-molecule fluorescence), is that they allow the sample to be analyzed under conditions that mimic the context of its native biological system [9]. Furthermore, the recent important development of single-molecule techniques, which allow us to study DNA–protein interactions one molecule at a time, significantly enhanced the effectiveness of microscopy-based approaches, allow visualizing the transient dynamics of molecular events.

### 2.1. Fluorescence Microscopy

Fluorescence is a physical property of some substances whose excitation by the absorption of an electromagnetic radiation with specific wave light leads to the promotion of an electronic transition, followed by a subsequent emission of a lower frequency light while the excited molecule returns to the ground state. The susceptible molecule to this process is also known as “fluorophore”. Each fluorophore is defined by a distinct absorbance and emission spectrum that makes it chromatically differentiable. Therefore, different fluorophores in the same sample can be simultaneously detected [10]. The first experiments in which the potential of fluorescence technology emerged were conducted by Rotman in 1961, through which enzyme β-D-galactosidase molecules in microscopic droplets were detected using a new fluorogenic substrate, the 6-hydroxyfluoran-β-D-galactopyranoside (6HFG) [11]. By then, the idea of using fluorescence as a detection tool in scientific techniques had been applied to research fields such as chemistry or basic physics; only in 1989 was it employed to observe single fluorescent molecules. Thereafter, several advancements have been achieved leading to the coupling of fluorescence and molecular biology for the investigation of complex biological systems thanks to its sensitivity and accuracy, the possibility of monitoring multiple fluorophores in parallel, and the ease of examining the results with several fluorescence microscopy techniques.

#### 2.1.1. Immunofluorescence

Immunofluorescence (IF) relies on the use of fluorescent-labeled antibodies which are employed to enable in situ imaging of specific protein cellular localization and organization by fluorescence microscopy. IF is a staining technique more sensitive than immunohistochemistry that offers signal amplification. There are two major IF approaches: direct (primary) immunofluorescence, that uses a single primary antibody conjugated to a fluorochrome binding directly to the target antigen, and indirect (secondary) immunofluorescence, in which the primary antibody binds the antigen while a second anti-primary antibody is conjugated with fluorochromes [12]. A recent example of the application of IF to demonstrate protein affinity towards specific DNA fragments is the work from Vasantha Niranjan et al., where the authors demonstrated how Laminin A and the telomeric repeat factor 2 (trf2) interact with DNA using fluorophore-labeled antibodies [13]. Trf2 is a protein associated with the Shelterin complex, interacting with the telomeric ends of DNA. In this study, Vasantha Niranjan et al. [13] used a novel approach for studying DNA–protein interactions by Antibody-Mediated Immunodetection. They used plates coated with specific telomeric DNA fragments, which were hybridized with a protein mixture. The target proteins bound to telomere sequences were identified by the corresponding antibodies and subsequently revealed with secondary antibodies conjugated with fluorophores. In the work, fluorescence data showed that both Laminin A and trf2 interact with specific DNA sequences. Furthermore, an immunoprecipitation assay was conducted to confirm specific protein–protein interactions.

Several studies about the spatial relationship between nucleic acids and associated proteins have been based on the use of IF, both in human cells and others biological organisms, such as Drosophila melanogaster [14]. M. A. Grande and colleagues’ work, for example, reports the use of IF labeling cultured cells to study the distribution and spatial relationship between the sites of newly synthesized RNA and the nuclear domains containing key transcription proteins, such as TFIIH, Oct1, and E2F1 [15]. Other studies in which this technique was exploited investigating transcription factor (TF) locations like FOXO in U2OS cell line [16], glucocorticoid receptors complexes, or the high dynamism of these proteins due to an external stimulus include drug treatments in rat cells [17]. IF has also been frequently used to evaluate how an external perturbation (like a stress or other external stimuli response) affects the translocation of various proteins, including TFs and nuclear receptors in different human and rat cell lines [18,19].

#### 2.1.2. Single Molecule Fluorescence

Small organic fluorophores can be bound to molecules of interest, and they can act as a detector to obtain important information about their localization in the investigated biological system. Currently, most detection techniques are based on large analysis that originates in a considerable amount of general data. However, novel emerging variants have gained widespread acceptance, such as single molecule fluorescence (SMF) that allows for the detecting of molecular subpopulations in complex mixtures. SMF enables the direct observation of elements of interested often overlooked by traditional bulk analysis, like molecular heterogeneity and real-time kinetic dynamics. Given its great potential to provide informative and qualitative data, SMF has been established as a detection technology on large RNA–protein assemblies to comprehensively study their binding or dissociation factors and their conformational heterogeneity. Accordingly, SMF hybridization studies are also performed to investigate dynamic and/or transient binding of two biomolecules. To that aim, the biomolecule of interest is attached to a specific surface and labeled with a fluorophore while its binding partner is labeled with a different one [10]. The great advantage of SMF technology lies in the ability to monitor a single molecule at a time thanks to a clearly distinct fluorescence signal. Various types of fluorescent dyes, such as CyDye, Alexa Fluor, and ATTO, are used in SMF. Their selection is based on characteristics such as brightness, photostability, and binding affinity to molecules. Their pH tolerance is important for labeling. Since the presence of oxygen causes photobleaching and may quench fluorescence, it is important to choose an oxygen removal system that does not interfere with the biological system under investigation. It may be useful to consult available RNA structural data, as RNA molecules exhibit non-standard pairings. Interactions with other molecules, as well as the internal interactions of the RNA itself and its conformational dynamics, must take this into account when choosing RNA sites to be labeled with fluorophores; in fact, labeling in a wrong site can block or alter the RNA’s function. If the RNA is less than 80 nucleotides long, then it can be synthesized chemically and the fluorophore can be inserted during synthesis or subsequently using reactive chemical groups. When the RNA is composed of multiple nucleotides, instead, enzymatic ligation is employed. Fluorescent proteins, such as GFP or mCherry, are often used for protein labeling with fluorophores. These can be genetically fused to the protein of interest. Given their large size, they tend to be used in in cell studies but not in in vitro ones, as they can interfere with the protein of interest and exhibit low brightness and blinking [20].

#### 2.1.3. Total Internal Reflection Fluorescence Microscopy

A selective illumination mode that aids signal visualization is total internal reflection fluorescence microscopy (TIRFM), which reduces background fluorescence illuminating only those molecules that lie closer to a selected surface. Unfortunately, TIRFM requires fluorophore concentrations of 100 nM, while many biological interactions occur at higher concentrations. In this case, a TIRFM variant named ZMW (Zero-Mode Waveguide) technology can be used [21]. ZMW technology involves a thin metal layer (aluminum or gold) containing nanoholes with a diameter of approximately 100 nanometers placed on a glass substrate; since the nanohole diameter is smaller than the light wavelength and hinders its normal propagation, light shone into the nanohole creates an evanescent field, with a confined illumination to a depth of only 20–30 nm. In this way, the evanescent field excites only one or a few molecules at a time, even in concentrated solutions of fluorophores [22].

Snead et al. [23] studied the ribonucleoprotein condensates of the glutamine-rich Whi3 protein through TIRFM. Whi3 can form large oligomeric structures with RNA (ribonucleoprotein complexes—RNPs) that can associate with the endoplasmic reticulum and grow until they are blocked. The authors demonstrated that membrane association controls condensate size and confirmed a trade-off between locally enhanced protein concentration at membranes and a reduction in diffusion, which reduces the increase; so, membranes allow the formation of condensates but limit their growth. Mekonnen et al. [24] used TIRFM to study FUS, a protein involved in RNA regulation. They studied the oligomerization state of mutant FUS in human neuroblastoma cells in amyotrophic lateral sclerosis (ALS), by combining TIRFM with Single-Molecule Pull-Down (SiMPull), aiming to identify the molecular mechanisms leading to the disease. Indeed, the FUS mutants associated with ALS lack of affinity towards RNA, thus preventing its normal dynamic remodeling because they form abnormal aggregates. TIRFM can also be combined with super-resolution microscopy techniques such as Stochastic Optical Reconstruction Microscopy, which produces extremely high-resolution images of protein complexes in living cells [25]. An alternative to TIRFM is Highly Inclined and Laminated Optical Sheet (HILO), in which the sample is illuminated with a thin and angled beam, allowing for the tracking of single molecules in living cells, essential for studying the RNP’s assembly and mobility [26]. Fluorescence cross-correlation spectroscopy (FCCS) instead may investigate the interaction between two molecules using two different colors of fluorophores [27]. In the study of cellular protein interactions, a limitation of TIRF is the penetration of the evanescent wave and the use of microscopes suitable for this technique in the experimental practice of laboratories. Xu et al. employed STORM also to study epigenomic transcriptional regulation elements, defining different chromatin structures through the functional dynamism of histone modifications in eukaryotic cells [28].

When RNA–protein interactions occur within biomolecular condensates, such as stress granules or nuclear bodies, they can be investigated using Fluorescence Recovery After Photobleaching (FRAP). FRAP measures how quickly a molecule becomes visible (fluorescent) in an area where it was previously invisible due to a laser action. This enables the study of its diffusion and molecular dynamics within a condensate. The FRAP technique is also applicable to the study of RNPs because it helps describe the protein or RNA mobility within the condensate and the binding dynamics; therefore, it provides indirect information on the interaction [24]. Huang et al. [29] studied the RNA-binding complex MRP1/2 in the mitochondria of Trypanosoma brucei, which consists of a single large mitochondrion. Direct and quantitative measurements made on living cells are necessary to understand the functions that occur in this environment. Therefore, they used FRAP to investigate the mitochondrial protein MRP1, which binds RNA, and measured the fraction of the complex that is mobile compared to the non-mobile fraction, both with and without mitochondrial RNA. Thus, they observed how the lack of mitochondrial RNA leads to an increase in the mobile fraction of the complex, defining that RNA binds and stabilizes part of the complex, decreasing its dynamics. Therefore, the FRAP technique allowed them to define how RNA is essential to slow down or block the protein and, consequently, how it regulates mitochondrial RNA metabolism. This application well reflects the potentiality of the FRAP technique to investigate transient interactions with spatial resolution. In fact, FRAP allows the identification of fluorescence recovery as unbleached, fluorescent molecules from the proximal areas are replaced and move into the bleached zone. Asamitsu et al. [30] studied the dynamics of the RNA-binding protein DNAPTP6, which interacts within ribonucleoprotein (RNP) and stress granules. Specifically, they investigated DNAPTP6′s affinity for RNA G-quadruplexes (rG4s) in neurons and whether this interaction is functional. FRAP allowed them to test the protein’s mobility within stress granules.

Parker et al. [31] have set up a specific protocol to study how RNA–protein interactions contribute to RNP granules’ structural organization revealing RNA visualization by single molecule fluorescence in situ hybridization (smFISH). This workflow may enable the study of protein–RNA, protein–protein, or RNA–RNA interactions’ role in stress granule structural configuration and potentially in any other intracellular membraneless organelle of interest. This protocol consists of several steps in which degradative enzymes, like nucleases and proteases, are introduced in living cells expressing G3BP1-GFP protein as a stress granule reporter through permeabilization. Choosing an appropriate marker is of key importance; i.e., G3BP1 or G3BP2 are organizing proteins involved in stress granule morphological arrangement. An additional critical step is the selection of a method to induce the expression of the protein marker of interest, e.g., create fluorescently labeled protein marker lines by transient transfection. The design of the smFISH probe will subsequently be required; it can be set up by free tools like Stellaris Probe Designer or Oligostan. In situ hybridization will allow the smFISH probe to bind a series of DNA complementary oligonucleotides fluorescently labeled to an RNA of interest which can subsequently be observed by microscopy.

TIRFM application can also be useful to improve DNA–protein interactions at a single molecule level through an innovative approach known as DNA curtains, in which DNA molecules are immobilized and aligned on the surface of a flow cell. Fluorescently labeled proteins are added and aligned with DNA filament, allowing for the DNA–protein interaction observation in real time. Gorman et al. employed the DNA curtains approach to uncover the dynamic and hierarchical mechanisms by which mismatch repair (MMR) proteins interact with specific regions containing DNA lesions [32].

#### 2.1.4. Fluorescent Resonance Energy Transfer

The most popular SMF techniques are those in which the single molecule approach is coupled with Förster or fluorescence resonance energy transfer (smFRET). The smFRET method relies on the energy transfer due to the close proximity between a fluorophore donor excited by a light source and an acceptor molecule that results as a fluorescence emission [33]. The FRET approach is a powerful technique to study molecular interactions; however, it suffers from the limitation of the mutual orientation between the donor and acceptor transition dipoles. It should be taken into consideration that FRET efficiency is at a maximum level when both dipoles are aligned, while it is at zero for perpendicular dipoles, regardless of their distance. Failure to consider the orientation of the dipoles could lead to the erroneous interpretation of FRET detection, and false negative results can occur even though they are actually at a nanometer distance [33,34].

Duran et al. [35] describe how SMF approaches like smFRET may be employed to investigate RNP-driven mechanisms within cellular extracts. smFRET allows for the study of RNA–protein interactions in vitro both with living cells and recombinant systems with limitations in the purification of some molecules or biochemical interactions not entirely faithful to environmental conditions, respectively. Therefore, smFRET combined with cell extracts in vitro experiments is an optimal single-molecule strategy for RNP characterization. Enders et al. [36] has implemented smFRET to study DEAH/RHA helicase Prp43 motility and the RNA duplex strand separation within RNPs remodeling. The DEAH/RHA family includes RNA helicases that conduct an ATP-dependent translocation in the 3′ to 5′ direction along single strands responsible for the RNPs’ dynamic organization during pre-mRNA splicing and ribosome biogenesis. G-patch factors are responsible for activating helicase activity by recruiting RecA domains during RNA catalysis and unwinding. smFRET experiments were used to follow ADP release from Prp43, fluorescently labeled with a mixture of Cy3-maleimeide and Cy5-maleimide, using TIRF microscopy. The authors had followed the G-patch and Prp43 activity through smFRET labels on the RNA substrate, thereby demonstrating how G-patch factors improve RNA binding and the rate of conformational transitions. In 2019, Jalihal and collaborators [37] described how the smFRET technique lends itself to the study of co-localization of single molecules or to the study of conformational changes that occur in the spliceosome within a distance range of 3–8 nm.

smFRET technology can also be applied to the study of RNPs, helping to understand how the RNA’s shape and interaction can change over time. These analyses are complex because each molecule can behave differently and the information changes depending on the system under study. In this case, selective labeling and enrichment approaches can be used. An interesting example is SiMPull, which relies on the selective isolation on an imaging surface of specific molecular complexes from cellular lysates to perform a single-molecule analysis. Blanco et al. [38] instead, developed a Single-Molecule Cluster Analysis (SiMCAn) to group molecules based on their FRET behavior, thus allowing to identify clusters of molecules generating similar complexes with RNAs.

smFRET also offers several advantages for DNA and protein interaction investigation. Indeed, its application provides critical insights into the complex processes of DNA association and dissociation. smFRET enables the study of how TFs are able to locate their specific target sequences on the DNA strand; Lee et al. used smFRET to study the transcription elongation dynamics, directly observing the structural changes of individual nucleosomes in real time and resolving the previously inaccessible dynamics of transcription elongation [39]. In recent work conducted by Paul et al. [40], they exploited smFRET to study the fundamental role of proteins that bind single-stranded DNA such as bacterial single-stranded DNA-binding proteins (SSBs) and eukaryotic replication Protein A (RPA) dynamic protagonists in genome stability, and other DNA processes as replication, repair, and recombination.

#### 2.1.5. Aptamers

RNA-FISH technology employs fluorescent probes to identify, locate, and bind to specific RNA target molecules within the cell; by observing the cell under a fluorescence microscope, it makes it possible to visualize the locations where RNA is present, even at the single-molecule level. This allows for the investigation of fundamental biological processes, such as gene expression and transcriptional regulation. MS2-GFP, on the other hand, is a real-time RNA imaging technique inside living cells that uses the viral MS2 sequence inserted into the RNA of interest and the viral MS2 coat protein fused to GFP; in this way, when the RNA is transcribed, the MS2-GFP protein binds to its MS2 sequences, making the RNA visible. Both FISH and MS2-GFP conjugation are two quite new methods that allow the detection and study of specific RNAs of interest and their interactions within the cell. They are highly sensitive technologies that enable the detection of a single RNA molecule, but they have some major limitations: FISH requires cell fixation, which limits it to static RNA imaging; the MS2-GFP system allows the RNA to be dynamically detected in living cells but suffers from high background fluorescence and requires genetic fusion modifications that could alter the RNA biological function. GFPs show several characteristics that have significantly improved the proteins in real-time study in living cells, such as their constitutive fluorescence and their ability to be produced by the cell itself, as they can be encoded by DNA. These properties have inspired the development of similar fluorescent reporters for RNA. In this case, the fluorescent reporter is a molecule specifically bound to a modified RNA which presents a structure called an “aptamer” that allows it to bind to the fluorophore. The RNA molecule can bind to the fluorophore after transcription, thus becoming visible even in living cells. These specific RNA aptamers are also called Fluorescent Light Aptamers (FLAPs). A technical limitation to the use of these compounds is that aptamers are selected in the laboratory using the SELEX in vitro technique (cfr. Section 5.2.2); therefore, they often do not perform as efficiently in living cells. For this reason, aptamers must be modified to improve them after selection in the laboratory. FLAPs have great potential as tools for analyzing RNA interactions. An example is represented by the Spinach aptamer, which emits green light, and the fluorescent protein mCherry that emits red light. These two can be employed in a FRET-like approach: if an interaction occurs between the two molecules, energy will be emitted. In the absence of fluorescence emission, quenching could also be evaluated; indeed, quenching is a reduction in the donor’s fluorescence, so if quenching occurs, then energy transfer is still occurring. Despite their great potential, FLAPs have low quantum yields and low extinction coefficients, thus there are several works in progress to develop new fluorophores and novel methods to identify aptamers with higher affinity. Improving FLAPs increases the possibility of investigating important biological processes through the interaction analysis between molecules [41].

#### 2.1.6. Molecular Fluorescence Complementation Systems (BiFC, TriFC)

Biomolecular interactions may be investigated by different molecular biosensor technologies, such as molecular beacon (MB), FRET, or molecular fluorescence complementation systems like Bimolecular Fluorescence Complementation (BiFC) and TriFC.

Protein fragment complementation technologies consist of several steps that provide the restoration of a functional protein through the interaction between their fusion partners. BiFC originated in the 2000s and was first reported by Ghosh et al. [42]. This technology is based on the split of a fluorescent protein at specific sites by gene splicing in order to originate two non-fluorescent fragments that are coupled with a pair of proteins of interest through gene fusion. Therefore, in case of interaction between the target protein pair, the two fragments may be in close proximity, and thus, restoring the fluorescent molecule. Several functional proteins are employed, like galactosidase, lactamase, or ubiquitin; among them, luciferase is the most suitable functional protein for live cell imaging since it directly emits luminescent and visible signals [42]. BiFC technology is regarded as a label-free technology, since the reaction pairs do not need to be added into the cell as they are already included within it. This represents an important advantage since it avoids the introduction of exogenous reporter molecules into the observed systems. Furthermore, BiFC signals can be detected with an inverted fluorescence microscope with high sensitivity due to a low background noise. These advantages make the BiFC technology suitable for a wide range of applications in several research contexts, such as the study of protein oligomerization, G protein-coupled receptors (GPCRs), and signal transduction, or virus–host interactions [43].

A key challenge in studying protein–RNA interactions consists in the optimization of protocols involving biochemical strategies to map these bindings also in living cells, given their importance in the post-transcriptional processes of gene expression. To that aim, a novel method building upon the BiFC one has been developed, known as Trimolecular Fluorescence Complementation (TriFC). TriFC allows for the visualization of RNA–protein interactions in living cells by combining BiFC and MS2 systems. TriFC involves one of the fluorescent protein fragments which is associated with a reporter mRNA by the known protein–RNA interaction, while the other complementing one is attached to an RBP; thus, in case of interaction between the RBP with the mRNA sequence of interest, the two fragments approach each other and the fluorescent protein is restored [44]. In this technique, Venus protein, a bright variant of GFP, is often used. The two halves of Venus are bound to two different proteins: the MS2 coat, which binds to a specific RNA sequence called the MS2-binding site, and the protein of interest, which will potentially interact with a particular mRNA. The RNA is engineered to contain both the sequence recognized by MS2 and that will attract the sequence that could bind the protein of interest. If this binding occurs, the two halves approach each other and assemble, giving rise to fluorescence. This will confirm that the protein has bound to the RNA at a specific site in the living cell. While TriFC is highly relevant for observing RNA-binding proteins in their native positions, it has the disadvantage of being nonspecific, unlike other systems. This issue could be overcome by analyzing purified components in vitro. Another drawback is the irreversible crosslinking between the MS2 protein and the RNA target [45].

Different protein interaction pairs could be employed to set up a TriFC system. Yin et al. [46] reported the use of a mCherry/Venus TriFC system to image RNA–protein interactions in parallel in the same live cells. This system involves mCherry cleaved into two fragments, bound to viral peptides (such as HIV TAT) and corresponding viral RNAs (RREs). After validating the system’s functionality, they used it to observe the interaction between the influenza A NS1 protein and the 5′UTR of various influenza viral mRNAs. Thus, the combined use of two TriFC systems (mCherry and Venus) allowed them to observe multiple RNA–protein interactions simultaneously in a single cell.

Huranova et al. [47] combined FRET technology with TriFC in an application known as RB-FRET, overcoming some of the limits of the two approaches. This technology is particularly useful for studying RNA-binding proteins with known RNA target sequences. In RB-FRET, the RNA-binding protein of interest contains a fluorescent protein as a donor, while the MS2 coat protein is labeled with another fluorescent protein and used as a FRET acceptor; then, an engineered RNA target contains both the binding site of the RBP of interest and the high-affinity MS2 site in close proximity to the former. When both proteins bind to the RNA, the two fluorescent proteins will be close enough to activate FRET. To measure FRET, the acceptor photobleaching technique is used: the acceptor is bleached with intense light so it can no longer receive energy. If FRET was in progress, then the donor is quenched and without the acceptor, it becomes more fluorescent. So, the fluorescence increase is evidence that FRET was occurring and that the two proteins were close together. As a control for the experiment, the same can be repeated with an RBP that does not bind to RNA; thus, no FRET should be observed to confirm that the previous signals were due to specific interactions [48].

### 2.2. Probe Microscopy

Atomic force microscopy (AFM) is a high-resolution microscopy technique that allows for the direct visualization of single molecules, oligomeric complexes between 1–10 nm, and even larger condensates up to 1 μm in size [24]. AFM was developed over 30 years ago and it has immediately become a fundamental tool for studying nanoscale biological structures. Its importance lies in the optical lever detection principle, that allows the use of AFM even in aqueous environments; thus, AFM can be employed in buffered physiological conditions that mimic those of the human cells. This represents an opportunity to study biological molecules in their native state, without sample labeling or staining, so it is suitable for protein–nucleic acid native environment interaction studies. However, it should be noted that a significant number of applications still involve the use of AFM not in solution but by fixing and drying the sample on the surface. For what concerns DNA samples, dehydration often induces a conformational change in the DNA from the B-DNA form to the compact and dehydrated A-DNA form [49]. Also, proteins are often destructured or distorted by dehydration and generally the protein molecular weight measured with AFM is lower than the actual one, due to the flattening procedure [50]. For this reason, there is a strong dependence between AFM volume and molecular weight for proteins smaller than 200 kDa.

Surface preparation for sample analysis is a critical step in studying protein–DNA interactions using AFM, as inadequate preparation could compromise the interaction between the scanning tip and the sample, resulting in imaging artifacts. Currently, one of the most widely used AFM substrates is mica, as it provides a flat atomic surface, is free of impurities, and is easy to prepare.

AFM uses a micro-lever, known as cantilever, able to exert a force ranging from 10 piconewtons (pN) to nanonewtons (nN) in the vertical direction on a single molecule bound between the probe tip and the sample surface. The deflection of the cantilever is directly related to the magnitude of the force applied to it. The tip interaction with the sample surface causes the cantilever deflection, which is typically detected by an optical system. Then, the cantilever height is adjusted by a feedback circuit to ensure that the system traces the surface topographic profile [51].

AFM has emerged from other technologies because it offers the advantages of obtaining detailed topographical nanoscale images, measuring forces in the picoNewton range, and operating in “tapping” mode. This enables it to touch the sample surface without damaging it, allowing for the study of structures such as double-stranded RNA (dsRNA), protein–RNA complexes, and nucleic acid aggregates in both nebulized and liquid conditions. AFM permits the analysis of molecular interactions between RNA and proteins, even at the single-molecule level, investigating the binding of the interacting molecules and their interaction physical nature [52]. Fuhrmann et al. [53] studied the interaction between the RBP AtGRP8 (Arabidopsis) and its target RNA; they bound the RNA to the AFM tip using PEG and immobilized the protein on the surface, observing two binding states with different characteristics and establishing the binding characteristics. Heus et al. [54] instead investigated the binding between the HIV Rev peptide and its RNA target, RRE; they bound Rev to the AFM tip and the RNA to the surface, also using two RNA mutants. Through AFM, they distinguished between the binding of the functional and non-functional mutants; then, they observed how the binding could be blocked by neomycin. Thus, AFM could also be employed to screen antiviral drugs that target RNA molecules. Tripepi et al. [55] describe a protocol for the visualization of RNA–protein complexes through AFM without the use of metal cations, such as Mg^2+^, which are often required for RNA or protein adhesion to surfaces. The proposed model describes the binding of RNA to the Staufen protein, an RBP involved in RNA transport and localization. It demonstrates how AFM allows for the visualization of RNA–protein complexes without the need for fluorescent labeling or other physiological modifications; it also allows for the characterization of mechanical properties such as the elasticity or rigidity of complexes.

AFM approaches also enable topographical images of protein–DNA to be obtained thanks to AFM’s high-resolution visualization of individual DNA molecules with proteins bound, enabling the complex size and protein number determination, their position on the specific DNA sequence, and the overall length of the DNA-protein complex. To determine the binding position, it is necessary to measure the DNA contour length from one of its ends to the midpoint of the protein attached to it [51]. The first measurement image is in pixels, and the values are reported as a percentage of the total DNA contour length or converted into nanometers (nm) or base pairs (bp) based on the image’s resolution. Then, individual binding events can be displayed in a histogram and fitted to a Gaussian distribution. The standard deviation of the resulting Gaussian distribution reflects the protein’s specificity for a given site-binding position which will show how DNA–protein interaction is specific, while proteins that do not specifically bind the DNA target are expected to be found randomly on double strands. The protein is much more likely to bind to the exact spot, when a specific target site is introduced [56]. Nettikadan and colleagues have utilized AFM to quantitatively analyze how the transcription factor AP2 binds to DNA, specifically mapping its interaction with the promoter sequence [57]. Chen and colleagues also specifically demonstrated how the binding of the base excision repair human 8-oxoguanine DNA glycosylase (OGG1) can probe DNA helix, searching for damaged bases, by inducing bends using AFM [58]. Many studies have been carried out in bacterial microorganisms using AFM regarding the mismatch repair pathway, focusing on essential proteins such as MutS that recognize the mismatch and the subsequent recruitment of MutL and MutH. For example, Wang and his colleagues used AFM to study MutS-induced DNA folding and its binding specificity [59].

### 2.3. Electron Microscopy

#### 2.3.1. Cryo-Electron Microscopy (Cryo-EM)

Cryo-electron microscopy (Cryo-EM) technology leads to the determination of the three-dimensional structure of biomacromolecules with near-atomic resolution and without crystallization. This technique uses a transmission electron microscope at cryogenic temperatures, through which biological molecules frozen in vitreous ice are observed. Microscopy collects thousands of two-dimensional images, which are then computationally reconstructed to obtain the three-dimensional structure of the molecule under investigation. As a technique, it offers numerous advantages, including the ability to work with minimal sample quantities (e.g., 1–5 μg/μL) and the possibility to visualize large molecules and molecular complexes, even >100 kDa. On the other hand, it is not suitable for smaller molecule analysis that results in a low signal; in these cases, it is advisable to bind the target protein to protein scaffolds, such as antibodies, to apparently increase their size and make them more visible.

Cryo-EM application in the study of the Dicer ribonuclease is well known; thanks to Cryo-EM, complex structural and functional information was defined [60]. Another important application of this approach dates back to the COVID-19 pandemic; through Cryo-EM, the structure of the SARS-CoV-2 replicative polymerase complexed with a template RNA duplex was determined. This was fundamental because the virus’s structure identification and its replication mechanism aided the rapid development of drugs that target the polymerase complex to inhibit viral replication [61]. Chenavier et al. [62] studied the influenza helical nucleocapsid structure by cryo-EM; it is a viral ribonucleoprotein complex consisting of viral RNA, nucleoprotein (NP), and trimeric polymerase. Cryo-EM allowed for the first time a direct and detailed visualization of NP–RNA and NP–NP interactions as well as the topology of the helical assembly.

He and colleagues used Cryo-EM to capture General Transcription Factor (GTF) image snapshots at different stages of assembly and to identify the position of each of them, and how each factor influences the assembly transcriptional complex [63]. This led to a series of other interesting discoveries about the transcription machinery key players, such as other co-factors according to research conducted by Plaschka and colleagues [64]. Despite these advances, some samples remain challenging even for Cryo-EM [65].

#### 2.3.2. Correlative Light and Electron Microscopy (CLEM)

The correlative light and electron microscopy (CLEM) enable information about specific protein functions and localizations in the cell to be obtained, by combining the power of fluorescence microscopy and the immuno-label’s ultrathin sections of fixed samples, with electron microscopy for the ultra-structural analysis of the same sample area. CLEM electron micrographs can be obtained using transmission electron microscopy (TEM) to visualize sample cross-sections from 50 to 100 nm with a very high resolution [66]. CLEM can be employed to study specific proteins of interest, such as TFs in the transcription process, DNA repair proteins, or the fluorescent Polycomb group (PcG) that are found to be diffusely distributed in cell nuclei, identified as nuclear domains enriched in separated heterochromatin fascicles, as described by Smigovà et al. studies [67]. Tonnemacher and colleagues [68] employed CLEM to investigate about cellular chromatin compaction and organization after external stimuli, like carbon or iron irradiation.

Another performing technology is the Cryo-CLEM, which combines the fluorescence microscopy and the electron microscopy advantages in cryogenic conditions to further maximize the benefits of both techniques. Cryo-CLEM allows for the direct correlation of molecular structural details with Angstrom precision [69] preserving the sample’s native state and ultra-structural vision; it was employed to study the membrane architecture of lamellar bodies (LBs) and surfactant-rich organelles found in lung alveolar cells, as seen in Klein et al.’s study [70].

## 3. Immunocapture Systems: RNA and Protein-Centric Methods

Investigations based on RNA–protein interactions are becoming increasingly interesting and the subject of extensive study. In addition to a variety of innovative techniques, the use of affinity-based immunoprecipitation methods is attracting considerable interest. Depending on whether the starting molecule of interest is RNA or protein, these methods are classified as RNA-centric and protein-centric. Protein-centric methods rely on protein immunoprecipitation resulting in the detection of co-purified RNAs, while RNA-centric methods aim to identify ribonucleoproteins (RBPs) capable of targeting a single RNA of interest.

### 3.1. Protein-Centric Approaches

Most protein-centric methods include several techniques based on the use of antibodies against specific proteins under native conditions, and are generically called immunoprecipitation (IP) techniques. These techniques are well suited for the characterization of both RNA and DNA interacting with proteins. RNA immunoprecipitation (RIP) is one of the first methods based on the isolation of a specific protein and the characterization of the interacting RNAs by sequencing (RIP-seq) or microarray (RIP-chip) [71]. Since the native conditions under which the technique is performed represent one of the main problems, they can provide a wide spectrum of non-specificity and possible artifacts. Therefore, several optimizations of these techniques have been implemented, leading to the development of different crosslinking immunoprecipitation (CLIP) techniques [72]. CLIP is based on the use of bifunctional compounds able to create a covalent bridge between residues interacting in the investigated complex, thus providing specific information on the contact points stabilizing an intermolecular interaction [73]. The use of highly efficient crosslinking strategies can reduce false negatives and improve time efficiency. Indeed, several CLIP-based techniques have been developed to improve the identification of RNA–protein interaction. These include high-throughput sequencing (HITS-CLIP), photoactivatable ribonucleoside-enhanced CLIP (PAR-CLIP), individual nucleotide-resolved CLIP (iCLIP), and enhanced crosslinking immunoprecipitation (eCLIP) [71,73]. Except for the final sequencing steps, they differ in the crosslink activation method and their applicability, which depends strictly upon the biological starting material. HITS-CLIP is a high-throughput sequencing-CLIP based on a bifunctional reagent that can be activated by UV light [73]. In this approach, once a cell lysate is obtained, the RNA is partially digested using RNase and the immunoprecipitation of the protein of interest is performed. Subsequently, a radiolabeled linker is then added to the 3′ end of co-immunoprecipitated RNA fragments using alkaline phosphatase. Addition of the linker to the 5′ end is avoided to prevent a high background signal. The resulting modified complex is then irradiated by UV light at a wavelength of 254 nm and thus generating covalent bonds between the RNA fragments and the protein residues involved in the interaction. As with most CLIP variants, samples are then resolved on SDS-PAGE, isolated from the membrane, and digested with proteinase K to prepare RNA fragments for reverse transcription and sequencing [72]. This variety of CLIP allows for the production of high-throughput sequencing reads without causing high levels of noise during analysis. Nevertheless, UV crosslinking has sometimes proven inefficient [74]. To overcome these limitations, PAR-CLIP was developed. Unlike HITS-CLIP, PAR-CLIP crosslinking agents are activated by visible light (λ = 365 nm). Subsequently, the modified nucleosides, 4-thiouridine (4-SU), and 6-thioguanosine (6-SG) are incorporated into nascent RNA during the transcription process. This CLIP variant has the advantage of providing highly specific identification of binding sites on RNA. This innovative application, based on the integration of 4-SU and 6-SG into RNA, allows to discriminate labeled from unlabeled RNA. However, the high cost of this approach and the need for in cell labeling limits its broad applicability [73,75]. Like PAR-CLIP, iCLIP enables the identification of RNA–protein crosslink sites at single-nucleotide resolution [76,77]. The method involves cell lysis, immunoprecipitation, the addition of an adapter to the 3′ end, and radioactive labeling of the 5′ end. Reverse transcription is followed by cDNA circularization, which is more efficient than traditional adapter ligation [78]. Although this approach involves many enzymatic and precipitation steps, it is widely applicable, even in experimental conditions such as animal tissue, where PAR-CLIP is not feasible. As an improvement on the iCLIP technology, eCLIP was finally developed, thus reducing the complexity of its implementation. Technically, after UV crosslinking, the canonical cell lysis steps, and RNase digestion, 3′ adapter ligation is performed, followed by proteinase K digestion. Reverse transcription is performed to obtain cDNA, to which DNA adapters are ligated at the 3′ end for identification. One of the main advantages of this approach is the use of non-radioactive markers.

Regarding DNA, the ChIP approach was developed to characterize DNA interacting with proteins. This technique allows for the identification of genomic binding sites of specific regulatory proteins [79]. Usually, proteins and DNA-binding sites are crosslinked, and then an antibody is used to isolate the target protein–DNA complexes, thus proceeding to analyze the deoxynucleotide sequences. Based on technical differences in sample preparation, several modifications were made, thus giving rise to the following techniques: chromatin immunoprecipitation sequencing (ChIP-seq), (CHIP-exo), (CUT&RNA), and (CUT&TAG). In ChIP-seq, the antibody is used to selectively recognize the target protein, thus allowing for the characterization of the DNA fragments bound to it. Formaldehyde is commonly used to fix proteins at their binding sites in their native conditions and the DNA fragments of interest are characterized by sequencing. In recent years, several variations of this technique were developed. For example, the identification of DNA fragments by hybridization with a microarray led to the development of a ChIP-chip technique [80]. Besides, ChIP-exo was developed to increase the sensitivity and resolution of protein–DNA interactions. It relies on the action of an exonuclease capable of digesting chromatin near the site of formaldehyde-induced protein–DNA crosslinking, thus achieving near-single-base resolution [79,81]. To ensure efficient high-resolution sequencing of libraries, ChIP was initially implemented using the so-called CUT&RUN (Cleavage Under Targets and Release Using Nuclease) technique. This involves the binding of a selected chromatin protein to a specific antibody. DNA is cut by a fusion protein consisting of a micrococcal nuclease (MNase) cutting domain and a protein domain (pA/G) capable of specifically binding the antibody [82]. Similarly, the CUT&Tag (Cleavage Under Targets and Tagmentation) technique is based on an enzymatic-anchoring strategy where MNase is replaced with the A-Tn5 transposase fusion protein. Activation of this protein allows for the generation of a high-resolution DNA library by inserting sequencing adapters into the cleaved DNA fragments. This technique ensures a higher signal-to-noise ratio [83] compared to other ChIP approaches.

### 3.2. RNA-Centric Approaches

RNA-centric approaches were developed to identify RBPs involved in binding to a specific RNA using mass spectrometry. These techniques rely on intracellular crosslinking followed by pull-down of the RNA of interest using short biotinylated oligonucleotides, thus allowing to isolate the investigated RNA and identify the proteins that interact with it. RNA-centric approaches include RNA antisense purification (RAP), chromatin isolation by RNA purification (CHIRP), and RNA capture target hybridization analysis (CHART). Although these techniques share a common goal and methodological framework, differences in probe design and technical complexity distinguish their practical applications [84]. RAP is a technique that allows the mapping of lncRNA interactions with chromatin [85]. It is widely used but designing biotinylated antisense oligonucleotides to target accessible regions of the RNA of interest is often challenging. These nucleotides are generally long and highly specific to the target region, thus avoiding any off-target interactions. One disadvantage of this approach is the need for large quantities of starting materials to allow for adequate mass spectrometry analysis. CHIRP-MS is a technique based on the use of tiling oligonucleotides that allow for the targeting of specific lincRNAs with bound protein and DNA sequences [86]. CHIRP-MS and RAP-MS were used to characterize the Xist interactome, an lncRNA involved in X chromosome inactivation [87]. Unlike RAP, tiling oligonucleotides are preferred in CHIRP since they are arranged in a series, thus tiling the entire target region. CHART is a hybridization-based technique that allows the mapping of genomic binding sites for endogenous RNAs, based on the use of a few targeted and optimized oligonucleotides [88]. This technique was used to characterize proteins interacting with the human long non-coding RNAs NEAT1 (nuclear enriched abundant transcript 1) and MALAT1 (metastasis-associated lung adenocarcinoma transcript 1) involved in binding to active chromatin sites [89]. However, for all these techniques, proteins and DNA interacting with RNA are analyzed by mass spectrometry and NGS, respectively. Compared to other RNA-based approaches, CHART ensures low background and high specificity.

## 4. Surface Plasmon Resonance to Study Protein–Nucleic Acid Interactions

Surface plasmon resonance (SPR) is an optical biosensor technique based on the excitation of surface plasmons, which are electron oscillations at the interface between a metal layer and a dielectric element that enables real time, label-free monitoring of biomolecular interactions. It consists of monitoring changes in the refractive index near the surface of a sensor chip, which occur when biomolecules bind to ligands immobilized on it [90,91,92]. The SPR technique is especially used in the study of protein–protein interactions, protein–DNA/RNA interactions, small molecule–macromolecule binding, antibody–antigen recognition, and in the study of kinetic profiling of drug candidates [90]. Binding and unbinding between the two partners are plotted in real time, enabling measuring kinetic parameters of the interaction (i.e., association rate constant k_on_ and dissociation rate constant k_off_) and the equilibrium dissociation constant (K_D_) = k_off_/k_on_ [93].

SPR is a powerful tool to analyze different interactions between nucleic acids and their different partners, such as transcription factors, DNA/RNA-binding proteins, aptamers, and histones, and to study CRISPR/Cas9 target binding kinetics [93,94,95]. Immobilization strategies for nucleic acids on the chip could be different. The most used strategy is the amine-modified oligonucleotides covalently attached to a carboxy dextran-derivatized chip [96]. Other strategies are biotinylated oligonucleotides captured on streptavidin-coated chips (SA chip) or hybridized duplex DNA or RNA strands [93,96,97]. For nucleic acids/protein systems, SPR analysis offers the possibility to study not only equilibrium binding affinities and k_on_ and k_off_, but also helping to understand binding mechanisms, conformational changes, and the effect of sequence or structural variants on binding dynamic. SPR is now well established in the nucleic acids/protein field, as documented in different reviews and articles [95,98,99], but the effective use of SPR in protein–DNA/RNA studies require careful attention to multiple methodological details, because nucleic acids bring their own complexities: electrostatics, salt dependence and counterion screening, polyelectrolyte effects, and possible structural heterogeneity. Electrostatics and salt dependence are two important points to focus on during protein/nucleic acid SPR studies. Because nucleic acids are highly charged polymers, protein–DNA interactions are strongly modulated by electrostatics and counterion release. These characteristics influence the kinetics observed by SPR. It is observed in literature that kinetic parameters obtained by SPR depend on salt concentration (ionic strength) and valence of ions. By varying salt, it can often distinguish whether association or dissociation steps involve the release or uptake of ions and thereby infer the various steps (e.g., if binding is a single-step or involves intermediates) [90]. The changes in salt can shift both k_on_ and k_off_ substantially, because binding often involves the displacement of ions from DNA phosphate backbone and favorable electrostatic interactions. Thus, SPR experiments with DNA should systematically vary ionic strength (e.g., NaCl or KCl concentration) to test whether binding kinetics obey simple trends, and whether fitting remains consistent over the salt series modifications [100,101]. Furthermore, surface density should be moderate, as excessive ligand immobilization can cause steric hindrance, diffusion limitations, and mass transport artifacts, whereas insufficient coverage may result in low signal intensity. Typically, a surface density of approximately 100–300 RU is considered appropriate for short oligonucleotides, although the optimal value depends on the experimental system and interaction kinetics [101].

Ligand orientation is another critical factor. The nucleic acid should be immobilized in a configuration that preserves accessibility of the binding site, for instance, through a flexible linker or by anchoring at a terminal region distant from the recognition sequence [101]. To minimize non-specific binding, residual reactive groups on the sensor surface should be quenched after immobilization (e.g., with ethanolamine). This step prevents unwanted protein adsorption and improves baseline stability [101]. Finally, regeneration conditions must be compatible with the chosen immobilization strategy, allowing efficient removal of the bound analyte without denaturing or detaching the nucleic acid ligand. Achieving this balance is essential to maintain ligand integrity and ensure reproducible sensor performance over multiple cycles [101].

A common issue during the study of the nucleic acid–protein interactions by SPR technique, is the rebinding: when analyte molecules dissociate, they can remain in the proximity of the surface and rebind before diffusing away, artificially slowing the observed dissociation (k_off_). This is especially problematic for tight interactions (very low k_off_). Strategies to reduce rebinding include a lower ligand density (so fewer nearby sites to rebind), or the use of a competitor molecule in the buffer during dissociation (to scavenge free analyte). or, finally, a short dissociation of windows or partial regeneration to reduce cumulative rebinding artifacts [101]. Another central experimental issue is the mass transport limitation. During the analysis, the analyte must diffuse from the bulk solution to the sensor surface; if diffusion is slower than the intrinsic binding kinetics, the observed association rate will be limited by the diffusion and not by the real molecular k_on_, leading to underestimation of k_on_ [101]. Regeneration of the surface, that consists of removing bound protein to reuse the immobilized ligand, is often essential for the experiment throughput and reproducibility. But regeneration conditions can degrade or displace the ligand itself, especially nucleic acids. These problems could occur because the regeneration buffers may include high salt, low pH, detergents (e.g., SDS), or combinations of them. The choice must be mild enough to preserve DNA integrity and orientation. It is important to monitor the baseline RU after each regeneration cycle and verify that the ligand remains active; it could be good practice to re-inject a standard concentration of analyte periodically. In some experiments, partial regeneration is preferred over strict conditions to preserve ligands [99].

One of the first examples of efficient use of SPR in studying protein–DNA interaction appeared more than 20 years ago and is from Leontiou et al. [94] who studied the Topoisomerase IIL binding to DNA by SPR. Three DNA substrates with different secondary structures were studied: a 40 bp oligonucleotide, a four-way junction, and a 189 bp bent DNA fragment. In this study, Leontiou et al. [94] immobilized 40-bp linear DNA, the 4-way junction (4wj), and the bent-DNA substrate on a SA chip, and flowed the human topoisomerase IIα and β on them. A very interesting finding was that the measured k_on_ and k_off_ (and thus K_D_) varied with DNA structure, revealing differences in binding kinetics across linear, junctional, and bent DNA forms. However, both K and L isoforms exhibited similar binding kinetics, with average equilibrium dissociation constants ranging between 1.4 and 2.9 nM. This study is a good template: low immobilization densities (e.g., 84 RU for linear DNA, 135 RU for junction DNA), high flow, and careful regeneration [94].

In another paper [102], the authors studied the binding between bacteriophages P2, P2, Hy dis, and WΦ and their operators. The structural genes of these bacteriophages show over 96% identity, but for the repressor’s identity, these ranged from 43 to 63%. Furthermore, the operators, which contain two directly repeated sequences, vary in sequence, length, location relative to the promoter, and spacing between the direct repeats. In this study, the in cell effects of the wild type and mutated operators on gene expression were compared; such a comparison was carried out by studying in real time the formation of the complexes between the repressors and their wild type or mutated operators. The authors used the SPR instrument, immobilizing ~200–300 RU of biotinylated operator DNA (wild type and mutants from the three different bacteriophages: P2, P2 Hy dis, and WΦ) on an SA chip. They used two flow rates, 30 and 2 µL/min, flowing the C proteins of phage P2, P2 Hy dis, and WΦ, that recognize direct repeats, termed half-sites, but the repeats differ in sequence, lengths, locations relative to the early promoters, and spacing. Because some sensorgrams exhibited di- or tri-phasic behavior, the authors extracted the fastest association and slowest dissociation phases for K_D_ estimation. SPR analysis showed a reduced association rate constant and an increased dissociation rate constant for P2 and WΦ operator mutants. The association rate constants of P2 Hy dis were too fast to be determined. The P2 Hy dis dissociation response curves were shown to be triphasic, while both P2 and WΦ C were biphasic. This confirms that the kinetics of complex formation and the nature of the complexes formed differ extensively between these very closely related phages. Notably, the authors combined EMSA (Electrophoretic Mobility Shift Assay)-based affinity estimates and SPR-based kinetics to cross-validate their findings, a good practice especially when SPR may introduce artifacts.

As previously emphasized, several issues may occur in performing SPR analyses of protein–nucleic acid complexes. Among the others, one problem particularly affecting these studies is the possible degradation of the immobilized ligand, particularly in the case of RNA, or lose its correct conformation. This manifests as a progressive decrease in the baseline level or in the binding capacity (lower RU during the time). Moreover, since protein–DNA interactions are particularly sensitive to ionic strength, binding kinetics may change drastically with small variations in salt concentration. It is therefore very important to validate SPR results with orthogonal methods. Where possible, confirm K_D_ or kinetic parameters using other techniques (e.g., EMSA, ITC, fluorescence anisotropy) [100,102].

## 5. Alternative RNA-Centric Capturing Techniques

In this section, a heterogeneous group of approaches for DNA/RNA–protein complexes capturing will be described. All these approaches share the stabilization of endogenous RNA or DNA–chromatin assemblies with other nucleic acids or proteins without relying on antibodies. In these cases, the capture of native molecules takes place by base pairing of nucleic acids or by in situ ligation and crosslinking achieved with different approaches. In fact, these groups of techniques are generally referred to as RNA-centric techniques, since they are generally designed to capture the nucleic acid partner of the interaction. In addition to protein factors, in recent years, long non-coding RNAs (lncRNA) have emerged as key transcriptional regulators that also act as bridging protein complexes and building interactions on chromatin at distant sites [103]. These groups of techniques are particularly useful to investigate the long-range interactions in chromatin mediated by both proteins and lncRNA.

Most of these techniques are based on crosslink–hybridization capture directly carried out into cells to fix native molecular interactions, followed by a capturing oligonucleotide conjugating with a target DNA or RNA and allowing a purification of the complexes using different elution strategies.

These approaches retain molecular context, scale from single target to omic readouts (MS and NGS), and are highly modular with respect to crosslinkers, probe/linker design, and washing/elution conditions [104,105,106,107].

### 5.1. Crosslink-Hybridization Capture Techniques

In crosslink-hybridization capture techniques, interactions occurring in living cells are studied. When cells are in the desired conditions, the first step always consists of a crosslinking reaction between nucleic acids and their partners, achieved using different agents, formaldehyde (FA), UV, or psoralen in some variants. The desired target could be either nuclear and associated to chromatin or an RNA acting in other cellular compartments. After crosslinking, cells are lysed and generally chromatin undergoes enzymatic digestion or sonication to solubilize the sample and maximize the recovery of desired crosslinked analytes. At this stage, biotinylated antisense probes hybridize to the target RNA or genomic binding sites to co-purify its associated proteins. Streptavidin beads are then used to capture the crosslinked complexes. Stringent washes are carried out to eliminate unspecific interactors and then a controlled elution is performed. Elution typically occurs through a gentle biotin release in addition to heat to free probe–RNA complexes and reverse crosslinks. However, the washing procedure and elution approach used depend on the specific technique implemented and are critical points to set-up to obtain reliable and reproducible results and eliminating a-specific interactions.

This group of techniques encompasses chromatin isolation by RNA purification (ChIRP), capture hybridization analysis of RNA targets (CHART), RNA antisense purification (RAP), and hybridization purification of RNA–protein complexes followed by MS (HyPR-MS), which are described in the following subparagraphs.

#### 5.1.1. Chromatine Isolation by RNA Purification (ChIRP)

ChIRP is used to purify some specific RNA targets and its associated chromatin or proteins. Specific RNA sequences can be considered as targets; alternatively, an area of interest on the genome could be screened for the binding of RNA interactors by using tiling pools of ~20-nt biotinylated DNA probes (“even/odd” split pools) on crosslinked cells. After the beads capturing and elution, both the isolation of nucleic acids and protein partners can be screened. Isolated genomic targets are identified through sequencing, (ChIRP-seq) and protein partners through mass spectrometry (ChIRP-MS). In recent works, ChIRP-MS was the technique of choice, specifically to determine the interaction partners of different lncRNAs, which are emerging as fundamental regulators in immune modulation and cancer progression. The lncRNA myocardial infarction-associated transcript (MIAT) plays a key role in CD8+ T-cell exhaustion and was already associated with JAK3 as an up-regulator of the JAK/STAT pathway. ODD and EVEN probe sets were designed to target MIAT simultaneously, and the transcription factor ETS1 was found as a major interactor. ChIRP-combined results allowed for MIAT to be defined as a transcriptional activator of JAK3 forming a trimeric complex with ETS1 and JAK3 promoter [108]. ChIRP was also used to investigate the mechanism of epigenetic modifications occurring in lncRNA. Wei et al. investigated the role of N6-methyladenosine (m6A) in hepatocellular carcinoma (HCC) progression. The WT form of the lncRNA CTC-297N7.9 was found to bind to CCL2 and CD47 promoters by ChIRP assay, while its methylated counterpart did not come out as an interactor. As confirmed by following the experiments, the authors found that only m6A-methylated CTC-297N7.9 repressed HCC metastasis though regulating macrophage phagocytosis and M2 polarization [109]. Also, circular RNAs (circRNAs) are pivotal regulators of gene expression in oncogenesis. In a recent work, circNF1, a circRNA generated from the circularization of the neurofibromatosis type 1 (NF1) gene, was found to act as dual regulator on PD-L1 level from transcriptional and post-translational point of view, boosting the levels of this protein in esophageal squamous cell carcinoma (ESCC). ChIRP allowed for the identification of ANXA1 protein as a main binder and functional modulator of circNF1 [110].

This approach allows for an in cell analysis of molecular interactions and is quite robust when many short probes can tile the RNA. However, the use of several probes raises the costs and renders more difficult the set-up of stringency control in washing and elution needed to limit off-target capture. Regarding this, temperature of incubation and washing, salt concentration, and eluent concentration are all aspects to carefully consider. Moreover, the inclusion of scrambled/tiling-mismatch controls is fundamental to discriminate real interactors [104].

#### 5.1.2. Capture Hybridization Analysis of RNA Targets (CHART)

The CHART approach is very similar to the precediong one and was firstly described in 2013 using Drosophila roX2 RNA [88]. In this case, an RNase H digestion step is introduced for the elution from streptavidin beads in preliminary assays. This approach helps to identify single-stranded RNA regions. Since RNAse H specifically degrades RNA when it is associated in double-strand DNA, its implement allows for the selective identification of the regions of RNA that are single stranded and allows the release of bound material [104,105,107]. This preliminary screening allows for reducing the probe number and improving specificity under the same crosslink conditions used for the pull-down. In fact, once the accessible RNA regions using the RNAse H assay are identified, the actual CHART probes can be designed. Therefore, this technique is highly specific towards accessible regions. However, it also leads to an increase in time and cost for RNAse H mapping and empirical probe selection. Moreover, it is important to perform the RNAse H mapping under the same crosslinking and buffer conditions as the intended capture and validate probe candidates by qPCR mini-captures before scaling [104]. CHART is mostly used to investigate the interaction between RNAs and interacting chromatin loci or DNA targets. However, protein partners can also be identified using this technique [111]. As an example, West et al. used CHART to define both chromatin-binding sites and protein interactors of the highly expressed human lncRNAs NEAT1 (nuclear-enriched abundant transcript 1) and MALAT1 (metastasis-associated lung adenocarcinoma transcript 1). NEAT1 and MALAT1 showed co-localization to different genetic loci by CHART-seq; in particular, they were localized in nuclear bodies in close proximity to each other, nuclear speckles and paraspeckles. However, variations in the transcriptional features altered the localization of NEAT1 on active chromatin sites. Different protein partners were identified for the two lncRNAs by CHART-MS, suggesting different functions. However, a significant part of the interacting proteins was also shared among the two lncRNAs, suggesting a cooperation mechanism in regulating nuclear organization around nuclear bodies [89].

#### 5.1.3. RNA Antisense Purification (RAP)

In RAP assay, long 90–120-nt biotinylated antisense probes are used for high-specificity capture and for the identification of RNAs and lnc-RNAs. In this case, UV 254 nm incubation is commonly used as a crosslinking approach allowing optimal crosslinking for direct protein contacts. The incubation with beads is then performed and after stringent washes, RAP-seq can be used to analyze by sequencing of the DNA interacting strands isolated and released after heat incubation. Otherwise, when the protein partners should be analyzed, the elution step typically implies the use of benzonase endonuclease that progressively digests both RNA and DNA, therefore releasing the crosslinked proteins [105,106,107]. RAP generally confers superior specificity and signal-to-background data, having high input and longer probes/cost on the counterpart. The use of quantitative MS (SILAC/label-free) with replicate-based cut-offs to discriminate true interactors is generally recommended [104,105]. One of the most representative studies of RAP application is involved on the characterization of the well-known lncRNA Xist, regulating the transcriptional silencing of one of the X chromosomes during the development of female mammals. For the first time in this work, several interacting proteins responsible for Xist silencing were identified using RAP in mouse embryonic stem cells (mESC). SAF-A (also known as HNRNPU), SHARP, SMRT, and LBR were identified as interactors necessary to recruit HDAC3, histone deacetylase, and silencing transcription across the X-chromosome. These proteins were mainly involved in chromatin and RNA remodeling pathways, but data highlighted that these interact with Xist in a two-step mode, reflecting the development stage from the pluripotent state to cell differentiation [87].

#### 5.1.4. HyPR-MS (Hybridization Purification Followed by Mass Spectrometry)

HyPR-MS is based on the use of two or three capture oligonucleotides distributed across the RNA of interest. Crosslinking and beads coupling is performed like in other crosslink-hybridization techniques described, but in this case, a different elution approach is used. A toehold-mediated release (or toehold-mediated strand displacement) with a toehold oligonucleotide is implemented to displace the binding of the target and capture RNAs and to elute the target complexes. The toehold-mediated release (or toehold-mediated strand displacement) is a non-enzymatic, isothermal process allowing the substitution of a DNA or RNA strand with another. Thus, a short, single-stranded DNA or RNA “toehold” is used to initiate a cascade of reactions in which a new strand displaces a previously hybridized strand. This strategy of programmed hybridization of complementary strands offers a reversible and controllable method of manipulating nucleic acid structures. It is at the basis of different DNA-based innovative nanodevices [112]. A significant advantage of this technique is that it enables multiplexed, sequential purification of several RNAs from a single sample with good specificity/efficiency, allowing it to achieve a clean elution. However, it also requires an accurate in silico structure prediction of the toehold oligonucleotides (e.g., Mfold) and small-scale pilot captures to pick optimal sites. Negative toehold RNAs and spike-in standards for MS normalization are also recommended, contributing to render this strategy quite expensive [113]. Also, HyPR-MS was implemented for the simultaneous and selective isolation of the interactomes of lncRNAs MALAT1, NEAT1, and NORAD. These analysis highlighted SRSF and PRPF proteins and others contributing to both known and previously undiscovered roles of each lncRNA [113]. DDX41 gene encoding the DEAD-box RNA helicase 41 protein (DDX41) has a central role in the control of hematopoiesis in hematopoietic stem and progenitor cells. In the paper published by Dai et al., the protein interactors of the lncRNA, Gas5 (growth-arrest-specific 5), previously correlated with DDX41, were identified by HyPR-MS. Five proteins were also validated as Gas5 interactors by RNA-IP and a DDX41-regulation of Gas5 was confirmed [114].

### 5.2. RNA-Targeting Systems

RNA-targeting systems are designed to isolate and manipulate specific RNAs and their associated proteins within living cells or tissues, without relying on antibodies. These methods are highly versatile, allowing for the spatial and temporal targeting of single or multiple RNA species. The key technologies include engineered aptamer systems that bind RNA and recruit coat proteins or other biotinylation tags. Similarly, the PAIR technique couples PNA probes with RNA-targeting proteins for efficient in cell capture. Additionally, CRISPR-based tools (dCas13/RCas9) allow for precise RNA targeting for both capture and visualization, with the added flexibility of controlling the dynamics of RNA–protein interactions. These systems combine targeting specificity, non-invasive editing, and live-cell utility, making them powerful complements to traditional hybridization-based methods [105,107,115]. MS2/PP7/BoxB systems are ideal for high-throughput RNA capture and protein labeling, but they require sufficient RNA abundance and stable guide expression. PAIR-based and dCas13/RCas9 systems are preferred for in cell dynamic interaction screening, also in multiplex configurations, and especially if live imaging is the preferred screening technique.

#### 5.2.1. MS2/PP7/BoxB Aptamer Systems for RNA Capture and Visualization

Some systems for RNA capture utilize RNA aptamers, the most used of which are MS2, PP7, BoxB. Aptamers are generally defined as short synthetic nucleic acid molecules (DNA or RNA) with a three-dimensional structure that specifically binds to a target molecule with high affinity. Thus, aptamers could be used as an alternative to antibodies for capturing. In the applications described in this subsection, aptamers are fused at the 5′ of an RNA of interest to serve as baits for the capture of RNA and its interactors or enabling visualization of the RNA dynamics in cells [115,116]. The protein or small molecule recognized by the aptamer are then used for fishing the target RNA from a cell lysate, by using beads or a solid-phase matrix. Alternatively, when the aptamer target conjugated with a fluorophore is added in cells after aptamer–RNA incubation, a live-cell imaging of the target RNA localization can be obtained. Finally, these systems could also be conjugated with the proximity labeling techniques described in paragraph 3. In this case, the enzyme used for labeling is fused to the aptamer target and allows for tagging the RNA-interacting partners after incubation.

MS2 is an RNA aptamer with a stem–loop structure, that is incorporated into the target RNA and binds with high affinity to MS2cp, a coat protein of an Escherichia coli bacteriophage. This protein is used for purification of the target RNA and for the in vitro or in cell isolation of RNA-binding proteins. Alternatively, the MS2cp protein is fused to a fluorescent tag for live-cell imaging. Even though other aptamer systems were subsequently developed, MS2 remains the most used aptamer tag for these applications; and it was also used to develop high-throughput screens for RNA-interacting proteins. As an example, the incPRINT (in cell protein–RNA interaction) method was described by Graindorge et al. [117]. It employs HEK293T cells, stably expressing a NanoLuc luciferase–MS2cp fusion protein. When the target RNA-MS2 is co-expressed, the luciferase partner fuses to it. Then, the protein partner to screen is co-expressed in the cells fused to the flag tag. After cell lysis, the target complexes are pulled down using multiwells coated with anti-flag antibody. If the target protein is an RNA interactor, it binds to the well and the luciferase signal is revealed [117].

The PP7 system, similar to MS2, uses the PP7 RNA aptamer stem–loop, which binds with high specificity to the *Pseudomonas aeruginosa* bacteriophage 7 coat protein PP7. Also, this system has been successfully used for RNA–protein interactions in single-molecule studies, and is useful for multi-color RNA tracking [115]. Finally, the specific binding between the N protein of bacteriophage lambda and the BoxB aptamer is used as an alternative system. This latter is used to tether proteins to specific RNAs or to study mRNA stability, localization, and translation. Leppek et al. described the optimization of another aptamer, S1 with a four-fold repeat showed an improved affinity for streptavidin. In this work, S1 was attached to the AU-rich element (ARE) of tumor necrosis factor alpha (TNFα). ARE-binding proteins were purified from cellular extracts and identified by mass spectrometry, allowing for novel RNA-BP to be found, such as Rbms1 and Roxan, that had previously not been associated with AREs [118].

The use of an aptamer-based tagging system allows for work in biological systems without genome modification requirement. In fact, the aptamer-linked RNA is exogenous and co-incubated in cell lysates or living cells. The administration of the aptamer-binding protein would then allow dynamic RNA visualization in cell or RNA and interactor’s isolation. Caveats of this application are above all those related to scarce efficiency of the technique when RNA is not in high abundance, but high concentration of RNAs should often be avoided to prevent toxicity. In some cases, the addition of multiple RNA aptamer sites would improve aptamer binding and recognition. Scrambled RNAs conjugated with the same aptamer sequence should be used to assess the background and control for off-target labeling [115,116].

#### 5.2.2. SELEX (Systematic Evolution of Ligands by Exponential Enrichment)

SELEX (Systematic Evolution of Ligands by Exponential Enrichment) is an experimental in vitro approach first described in 1990. It allows for the selection of aptamers, short fragments of single-stranded DNA (ssDNA) or RNA folded into three-dimensional structures which enable them to selectively recognize and bind to a specific target. The SELEX method starts from large RNA or DNA libraries in which random sequences of 10–12 nucleotides are synthesized and subsequently incubated in the presence of the target ligand. The bound sequences are isolated and amplified by PCR; then, the cycle is repeated several times (typically 12 to 15 times) to progressively enrich the high-affinity sequences. Therefore, SELEX relies on several rounds of selection, to isolate the DNA or RNA sequences that are performing most in their binding characteristics towards target molecules. Both DNA and RNA SELEX can be employed. In the former, ssDNA is used (separating dsDNA resulting by PCR); in the second case, dsDNA is proceeded by T7 RNA polymerase enzyme to transcribe the DNA into a single-stranded RNA [119]. SELEX stands out for several key strengths, including aptamers’ robust structure (they can tolerate denaturing agents while maintaining their shape and function), and ease of library generation and automated procedures which help to reduce manual work errors. However, some disadvantages may be related to the potential alteration of the aptamer’s secondary structure, which reduces its effectiveness and can lead to false negatives or PCR-related errors [120].

Lin et al. [121] designed aptamers that specifically recognize the peptide–major histocompatibility complex (MHC) on cells, identifying two specific aptamers for cells expressing an ovalbumin alloantigen through a proof of concept and evaluating their potential future application in the selection of anti-tumor aptamers. Tsai et al. 2024, [122] employed SELEX to select aptamers which specifically bind to the Podoplanin protein (present on the surface of various tumor cells), in order to prevent its interaction with the platelet receptor CLEC-2, to block tumor platelet aggregation and reduce the tumor cells’ metastatic potential.

In recent years, SELEX approaches have evolved into high-throughput techniques, such as SEQRS (High-Throughput Sequencing of RNA Substrate Selectivity Landscapes) and HTR-SELEX (High-Throughput RNA-SELEX), automated methods that combine the SELEX principle with high-throughput sequencing (NGS). These technologies overcome the main limitation of studying only one or a limited number of RNA sequences at a time, simultaneously investigating thousands or millions of different RNA sequences and evaluating which ones bind optimally to the protein of interest. Thus, this experimental approach allows for an infinite global map of the proteins under investigation to be obtained [123].

#### 5.2.3. PNA-Assisted RNA Capture (PAIR)

PAIR (Peptide Nucleic Acid-assisted RNA Interactions) uses PNA probes. These are small, synthetic molecules with a neutral polyamide backbone and attached nucleobases, mimicking the backbone of DNA and specifically suited to hybridize with DNA and RNA targets. The absence of a negative charge on the PNA backbone confers high affinity and a strong and stable interaction with target nucleic acid. These probes, often tagged with biotin, facilitate the capture of RNA-binding proteins (RBPs) without the need for RNA crosslinking. PNA probes are generally designed to target a specific mRNA or lncRNA. The PNA probes can be further modified to include cell-penetrating peptides (CPPs) for in cell applications. The CPPs, with their superior efficiency in crossing cell membranes, are often used to facilitate efficient delivery of the PNA into living cells. Inside the cell, the PNA hybridizes to its target RNA and recipient cells are lysed and PNA-RNA-RBP complexes can be captured using magnetic beads coupled to a sense oligonucleotide complementary to the PNA. When the PAIR technique is used to isolate protein partners, a photoactivatable linker (Bpa) can be added to PNA. In this case, cells are exposed to UV light after the incubation with PNA, the Bpa is activated, and PNA forms covalent bonds with the RNA-binding proteins that are in close proximity to the target RNA. Therefore, this approach allows for the freezing of protein–RNA interactions to be achieved without the need of crosslinking steps, rendering it more efficient in studying dynamic interactions. In the pioneer work published by Zielinsky et al., PAIR technique was firstly implemented targeting 3′- and 5′-UTRs regions of the ankylosis mRNA with antisense PNAs transported into cortical neurons with the aid of a specific CPP. UV crosslinking allowed for the identification of different RBP proteins complexed with the target mRNA. IP in RNA–protein complexes was finally used to validate the screened interactions [124]. This work provided experimental evidence that RBP-mRNA interactions can be regulated by growth factor modulation.

Conversely, the PNA probes have high costs and require careful design for target affinity and sensitivity [116]. It is advisable to use short PNA probes that specifically target RNA motifs without secondary structure interference and to assess probe affinity by RT-qPCR before proceeding to large-scale capture.

#### 5.2.4. CRISPR-Cas-Based RNA Targeting and Visualization (dCas13/RCas9)

CRISPR-Cas, the largely known technology for gene editing, can be carried out using many variants of the Cas endonuclease. dCas13 and RCas9 are engineered CRISPR systems that operate on RNA and allow RNA targeting. The dCas13 system is a deactivated version of the Cas13 enzyme, which binds to RNA without cleaving it; thus, it could be used to target defined RNA sequences. If conjugated to biotin ligases, it would allow biotin tagging of interacting proteins, as demonstrated by Li et al. in their work, firstly describing this approach as CRISPR-based RNA proximity proteomics (CBRPP) [125]. The RCas9 system [126] more recently developed bears a fluorescent reporter fused to the dCas9, deactivated version of Cas9 endonuclease, and is used for real-time RNA visualization and live-cell imaging [115]. Alternative fluorescent proteins for multi-color live imaging have been reported. One of the first works reporting the use of CRISPR-modified versions to explore RNA–protein interactions was described by Yi et al. This method is defined as the CRISPR-assisted RNA–protein interaction detection method (CARPID) and was used to explore lncRNAs XIST interactors in the native cellular context [127].

Also in this case, one of the main advantages of the technique, in addition to the high specificity of RNA target recognition, is the efficiency of RNA interaction mapping without genome editing requirements. However, multisite guides often are necessary for efficient tagging, and accessibility and RNA secondary structures can hinder dCas13 binding efficiency.

### 5.3. Enzyme/Proximity Labeling Systems (TRIBE, RaPID, BioID/TurboID/APEX)

Enzyme-based proximity labeling techniques enable the identification and capture of proteins that are spatially proximal to a target RNA or nucleic acid complex without the need for crosslinking. These techniques rely on engineered enzymes (e.g., biotin ligases or peroxidases) that catalytically label nearby proteins with small molecules (e.g., biotin or other tags), which can then be captured and identified by mass spectrometry or immunoprecipitation. Labeling usually takes place on primary ammine groups. TRIBE, RaPID, BioID, TurboID, and APEX are key techniques that allow the high resolution of proximity labeling (<20 nm distance). These approaches are also applicable for the mapping of dynamic and transient interactions in cell and in their native cellular environments. Moreover, once the modification on the target protein is incorporated, the necessity to preserve the interactions of interest prior cell lysis is overcome in these approaches, and target purification can also be carried out in denaturing conditions [106,107,128,129]. In this paragraph, the main enzyme/proximity labeling systems are described. These techniques could be generally conjugated either with aptamer-based or CRISPR-based RNA targeting systems to direct the labeling enzyme to the site of interest.

#### 5.3.1. TRIBE (Targeted RNA Interactions and Binding Evaluator)

TRIBE exploits the ADAR (adenosine deaminase acting on RNA) enzyme to catalytically convert adenosine residues in the target RNA into inosine, detectable through RNA sequencing, which is the detection technique used in this case. This editing event serves as a molecular signature that marks RNA sites which were bound to specific proteins of interest. A key aspect of the technique resides in fusing the RBP of interest with the ADAR catalytic domain. After the interaction with target RNA, ADAR marks the target transcripts with inosine. Thus, TRIBE allows a direct and detectable tagging of RNA–protein complexes without the need for crosslinking, thus providing a clean readout of in cell binding on the nucleic acid. The density of editing sites can also be used to map the spatial organization of the RNA. However, it requires the assembly of fusion proteins and their selected expression in recipient cells. In addition, an adenosine proximal to the RBP binding site should be present, and the catalytic domain of ADAR has a strong preference for double-stranded RNA. For this reason, optimized ADAR constructs were developed to also achieve high editing efficiency towards single-stranded RNA. Biswas et al. described an experimental comparison between a crosslinking technique, CLIP, and TRIBE towards the β-actin mRNA. The study highlighted that the CLIP technique resulted in many false-positive proteins identified, while the implement of the TRIBE and RNA editing approach allowed for more specific results to be obtained, also allowing a clearer indication of the spatial organization of the RNA–protein interactions [130]. More recently, the ADAR2 enzyme was implemented in the so-defined HyperTRIBE. This approach was applied in Saccharomyces cerevisiae yeast living cells expressing two RBPs (KHD1 and BFR1) conjugated to ADAR2. In this case, the target transcripts were labeled by the enzyme and identified by high-throughput sequencing with high sensitivity and low background a-specific signals [131].

#### 5.3.2. BioID and TurboID (Proximity Biotinylation)

BioID and TurboID are proximity labeling systems to detect protein–protein interactions based on the use of biotin ligases to label proteins in proximity to a target RNA or protein. These systems utilize biotin ligase (e.g., BirA R118G) fused to a protein of interest or RNA-binding protein to catalyze the covalent attachment of biotin to nearby proteins. The biotinylated proteins are subsequently purified and identified via mass spectrometry, which is the preferred detection technique in this case. In a pioneer study on BioID set-up, Mukherjee et al. described the β-actin mRNA interactome and its changes of localization. Sixty additional β-actin–associated RBPs were identified by RNA-BioID and their dynamic interplay was unraveled [132]. BioID has also been extensively used to identify protein–protein interactions, such as the interactions of proteins of the mitochondrial matrix, such as ClpP mitochondrial protease, or E-cadherin, or centrosome structural proteins, and cytoskeletal proteins [132,133,134,135]. The authors in these cases produced the recombinant proteins of interest fused to BirA and different interacting partners were identified by LC-MS.

The TurboID variant offers an enhanced labeling efficiency, enabling reduced times (minutes to hours) and more precise proximity mapping. These systems were developed in the later 2010s [136] and are particularly valuable for mapping RNA–protein interactions in their native context [137]. An interesting example using TurboID involved the study of germ granules in C. elegans. These are aggregate regions condensing RNA and proteins and playing crucial roles in RNA metabolism and post-transcriptional gene regulation. TurboID proximity labeling technology allowed to identify two of the proteins that initiate germ granule formation, especially the part devoted to siRNA production [138].

However, these approaches give a strong background signal if the proximity radius is too large, and to optimize biotinylation efficiency, optimal fusion protein expression must be fine-tuned. High levels of the fusion proteins are required anyway and, thus HEK293, or HeLa cells, are preferred in this case, allowing for high recombinant expression. This aspect significantly limits the applicability of the technique.

#### 5.3.3. RaPID (RNA-Proximal Interactions Detected)

The RaPID method combines an RNA-targeting system, using BoxB aptamer, with BioID. In fact, it uses a λ-N (lambda N) peptide fused to a biotin ligase (e.g., BirA, BioID, or BASU) to bind specifically to RNA via the BoxB aptamer, which is inserted into the target RNA. The λ-N–Biotin ligase fusion covalently labels proteins that are within 20 nm of the target RNA, enabling protein isolation and identification by mass spectrometry [106]. RaPID allows a higher spatial precision for protein labeling compared to BioID, is capable of mapping dynamic interactions in real time, and does not require crosslinking. However, in this case, both fusion biotin ligase and modified target RNA are needed in different steps. Generally, the RNA of interest is recombinantly expressed in the cells and this could introduce artifacts in the experimental settings. Moreover, high-copy expression vectors are usually required to maximize biotinylation efficiency, and the BoxB motif should be placed in a sterically accessible region of the RNA [107,129]. RaPID is still a technique under development, but Ramanathan et al. described its potential in different applications, ranging from variation in protein interaction studies of aberrant RNAs present in genetic disorders to uncovering post-transcriptional networks in cancer models, and main proteic interactors of Zika virus RNA [139].

#### 5.3.4. APEX (Avidity-Based Protein Enrichment)

The APEX method utilizes engineered peroxidases, e.g., APEX2 (Apurinic/Apyrimidinic Endodeoxyribonuclease 2), as tagging enzymes. These catalyze the conversion of a phenol conjugated to biotin into a highly reactive biotinylating agent upon exposure to hydrogen peroxide. The product obtained links with high efficiency to electron-rich amino acids like tyrosine and lysine of proteins in proximity to the RNA of interest, and biotinylated proteins are isolated by streptavidin affinity purification. The APEX-seq method extends this to RNA-specific labeling and subsequent RNA–protein interaction mapping by next-generation sequencing [128,138]. This technique offers the most rapid labeling procedure (within minutes), is non-invasive, and can be performed in living cells. Lin et al. described the use of a CRISPR-based RNA tagging system; dCas13, conjugated with APEX. The U1 RNA of the small nuclear ribonucleoprotein complex was used as the target. The work identified proteins involved in both U1 canonical and noncanonical functions. The authors also described a plethora of poly(A) tail proximal proteins in a mammalian cell system [140].

Han et al. implemented APEX to determine the protein interactome of the human telomerase RNA (hTR), which regulates telomer transcription and has a central role in senescence. The authors compared an MS2 aptamer-based and CRISPR-based RNA targeting techniques to the hTR RNA, conjugating both with the APEX approach. The ALKBH5 demethylase enzyme was identified and validated as an interactor which modulates telomerase complex assembly and activity [141]. Even though most of the proteins identified were in common, analysis also revealed proteins unique to each dataset, remarking the importance of using complementary approaches. In fact, the different tagging approaches could be more efficient in labeling some neighbor proteins more than others, depending on the slightly different subregions targeted by each method [141].

On the other hand, limitation in APEX techniques comprises the requirement of a precise localization of the APEX-tagged RNA-binding protein, careful timing to avoid non-specific labeling, and high expression for optimal labeling efficiency. Therefore, pulse-labeling with short incubation times should be used.

## 6. X-Ray Crystallography

Crystallography has historically been the oldest method for studying the biomolecule structures and protein–nucleic acid complexes with atomic resolution. A beam of rays (or electrons) is diffracted by a crystal (an ordered aggregate of molecules), so the resulting diffraction pattern depends on the atom arrangement in the crystal; therefore, it helps reconstruct the molecule’s three-dimensional structure. However, the critical point in the process is represented by the crystal formation, as it is a complex process influenced by chemical and physical factors such as pH, temperature, concentration, and ionic strength. This can be done by reducing the protein’s solubility, thus helping the molecules join together to form the crystal, altering the water content, or using nucleation additives that bind to the protein to stabilize its shape or to break unwanted bonds (e.g., protein-solvent).

In 1953, X-ray crystallography enabled the acquisition of the first DNA double helix image by Watson and Crick [142]; from that moment on, a series of important discoveries have followed one another and contributed to a better understanding the transcription mechanism. In 2003, Reményi et al. [143] studied Oct4 and SOX2 structures through X-ray crystallography, and described how these TFs interact with DNA and other proteins. Protein–nucleic acid complexes crystallization presents intrinsic difficulties given their stability, which depends on specific experimental conditions, on the complex components, and the buffer composition. Typically, the protein and nucleic acid are mixed in a molar ratio of 1:1.2–1.5. It must be considered that some hydrogens are invisible during X-ray diffraction and can only be seen at high resolution because X-rays interact with electrons [60].

## 7. Nuclear Magnetic Resonance (NMR)

The nuclear magnetic resonance (NMR) technique allows for the obtaining of atomic-resolution structures based on the behavior of atomic nuclei placed within a static magnetic field and then perturbed by an oscillating field; the nuclei will respond to this field by emitting a signal of a specific frequency that depends on the nucleus type and the chemical environment in which it is present. The most common NMR techniques include 1H-NMR and 13C-NMR. There are two main limitations: one is that only nuclei with non-zero spin are active in NMR, so they must have an odd number of protons and/or neutrons; secondly, 12C, the most abundant isotope of carbon is not active in NMR, so molecules must be enriched with active isotopes to be studied. In the study of protein–nucleic acid complexes, both must be isotype-labeled or it can be decided to selectively label one of the two partners, so as to observe only the desired signal. Interactions at high concentrations can cause precipitation of the complex because they are highly electrostatic, since nucleic acids have negatively charged phosphate groups and proteins have positively charged regions. Precipitation of the complex can be avoided by increasing the salt concentration of the buffer. NMR spectroscopy can be combined with small-angle scattering (SAS) to study protein–RNA complexes with molecular weights > 50 kDa, as was done to study the archaeal ribonucleoprotein Box C/D complex bound to RNA (390 kDa). NMR spectroscopy can also measure translational diffusion, i.e., the molecule’s movement down a concentration gradient, to study the oligomerization dynamics and molecular complex formation [60].

Eladl Omar [144] reported a first study using NMR inside live human cells to observe the formation of an RNA–protein complex de novo. They employed a model system involving the HIV-1 Tat protein and its high-affinity RNA aptamer; Tat protein was expressed in HeLa cells and the aptamer was introduced into the cells by electroporation. This allowed them to directly observe the complex formation inside the cells and confirmed that RNA and protein were together inside the cell by confocal microscopy. Thus, the study has confirmed in real time the formation of a native RNA–protein complex inside live human cells, offering new possibilities for studying RNA interactions and describing how they regulate cellular processes under real-world conditions.

## 8. Conclusions

Protein–nucleic acid interactions are fundamental to the proper functioning of every organism, playing a pivotal role in most of the processes essential for cellular life. DNA–protein interactions regulate access to genetic information, such as transcription, DNA repair mechanisms, DNA physical conformation in chromatin organization, and replication. DNA-binding proteins have been discovered that dynamically assemble on the DNA double helix to form ribonucleoprotein complexes, translocate, and change their conformation in response to cellular metabolic and physiological dynamics. On the other hand, the numerous functions performed by different types of RNA are largely mediated by proteins that protect, transport, catalyze maturation and splicing, and control the binding with the partners of these nucleic acids. Gene expression control represents a key regulatory process in the flow from the genome information to functional proteins and the bulk of this regulatory activity is mediated by RNA-binding proteins.

**Figure 1:**
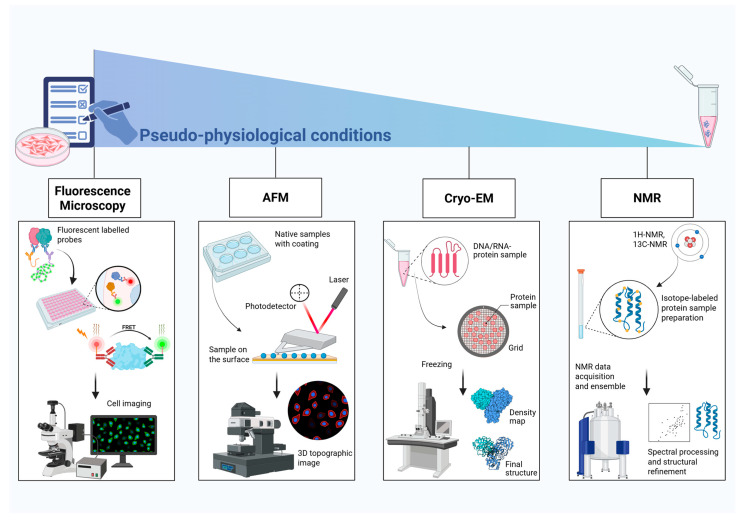
Schematic representation of the experimental procedures used for the microscopy-based and spectroscopy-based techniques to determine protein–nucleic acid interactions. The techniques are ordered according to their potential to preserve the binding under pseudo-physiological conditions. Created in https://BioRender.com.

**Figure 2:**
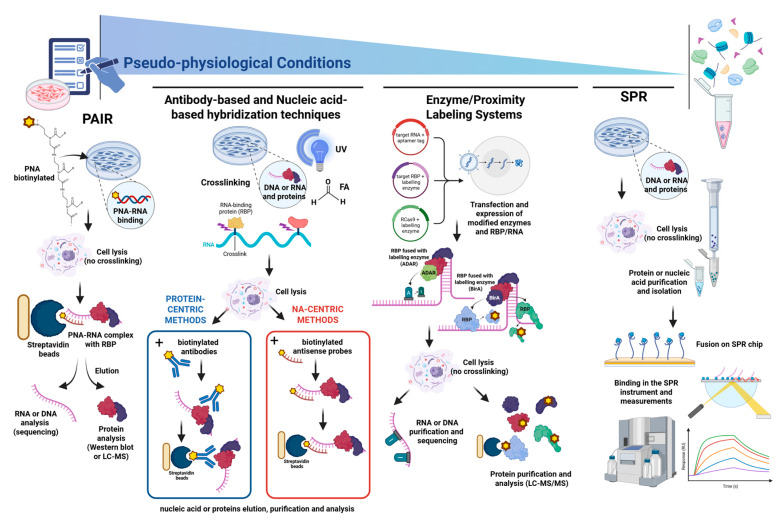
Schematic representation of the experimental procedures used for the capturing of the interaction complexes of proteins and nucleic acids. Data analysis is subsequently performed by mass spectrometry or Western blot for proteins and sequencing or RT-PCR for nucleic acids. Surface plasmon resonance allows for the observation of the binding between purified components in vitro and to determine binding affinity. The techniques are ordered according to their potential to preserve the binding under pseudo-physiological conditions. NA (Nucleic Acid), PNA (Peptide Nucleic Acid), PAIR (PNA assisted RNA Capture), RBP (RNA-binding protein), FA (formaldehyde), UV (ultra-violet), ADAR (Adenosine Deaminase acting on RNA), BirA (Biotin Ligase), SPR (Surface Plasmon Resonance). Created in https://BioRender.com.

Studying and understanding the protein–nucleic acid binding in detail is made possible by new technologies that allow us to visualize the multidimensionality of these activities, as summarized in Figure 1 and Figure 2, and Table 1 and Table 2. These technologies provide mechanistic insights into the biomolecular processes of cellular metabolism and, therefore, are key to understanding their application in the development of potential new therapeutic strategies and cellular biology.

## Figures and Tables

**Table 1:** Summary of major microscopy and structural biology techniques used to study protein–nucleic acid interactions reported in this review.

Technique	Main Advantages	Main Disadvantages	Resolution	Applicability	Quantity of Sample	Costs	Paragraphs
Fluorescence microscopy (incl. IF, TriFC, RB-FRET)	Allows imaging of molecules in their native biological context. Enables single-molecule visualization, multi-color detection, and real-time tracking. TriFC and RB-FRET permit dynamic monitoring of RNA–protein interactions in living cells.	Requires fluorescent labeling. Risk of non-specific signals (e.g., TriFC irreversible binding). Limited ability to define precise nucleotide-level contacts.	Low to medium resolution; detects proximity or co-localization, but not precise atomic contacts	Both in cells and in vitro	Low-moderate; depends on labeling strategy and fluorophores	Moderate; requires fluorescence microscopes, sometimes FRET-capable systems	Section 2.1 and Section 2.1.6
Atomic force microscopy (AFM)	Provides nanoscale topographical imaging (1–10 nm). Operates in physiological buffer, preserving native conformations. Enables single-molecule interaction analysis, mapping binding position along DNA/RNA. No need for fluorescent labeling.	Requires immobilization on surfaces. Binding measurements influenced by surface effects. Resolution limited for very small proteins or RNAs.	High (few nm); can determine binding position along DNA by contour-length measurements	Mainly in in vitro conditions	Very low sample amount per scan	Moderate; AFM instruments are specialized but not as costly as EM	Section 2.2
Cryo-Electron Microscopy (Cryo-EM)	Provides near-atomic 3D structures without need for crystallization. Works with minimal sample quantities (1–5 μg/μL). Ideal for large complexes (>100 kDa).	Poor signal for small complexes (<50–150 kDa). Requires sample vitrification and computational reconstruction.	High resolution (<5 Å), approaching atomic for suitable samples	In in vitro (in frozen hydrated samples)	Very low-moderate	Very high; expensive equipment and computational resources	Section 2.3.1
Correlative light and electron Microscopy (CLEM/Cryo-CLEM)	Combines fluorescence spatial specificity with ultrastructural EM detail. Enables localization of proteins or complexes directly in cells. Cryo-CLEM preserves native structures with high precision.	Requires specialized workflows. Fixed or cryogenic samples (no live imaging in EM).	High resolution, down to nanometer or sub-nanometer range (via EM)	In in cells (samples are fixed or cryo-frozen	Low-moderate	High; requires both fluorescence and EM platforms	Section 2.3.2
X-ray crystallography	Provides highest atomic resolution (<2 Å). Extremely detailed structural information about protein–DNA/RNA complexes.	Requires high-quality crystals, often difficult to obtain for nucleic acid complexes. Yields static snapshots of dynamic complexes.	Atomic resolution (<2 Å)	In in vitro (samples are crystallized and frozen)	Moderate-high; depends on crystallization success	High; requires crystallography facility and often synchrotron access	Section 6
Nuclear magnetic resonance (NMR)	Atomic-resolution structures in solution. Captures dynamic interactions and multiple conformational states. Allows selective isotope labeling of RNA or protein	Requires isotope labeling. Limited to <30–50 kDa complexes unless advanced methods are used. Needs high protein and RNA solubility.	Atomic resolution (<2 Å)	in vitro	High sample concentration required	Very high; spectrometers and isotope-labeled samples are costly	Section 7

**Table 2:** Summary of biochemical capture and labeling techniques reported in this review.

Technique	Main Advantages	Main Disadvantages	Resolution	Applicability	Quantity of Sample	Costs	Paragraphs
Aptamer-based tagging (MS2, TriFC, RB-FRET, PP7)	Allows visualization and detection of specific RNA–protein interactions. TriFC/RB-FRET permit live-cell monitoring.	Requires genetic engineering of RNA. TriFC can be non-specific and irreversible.	Low-medium resolution; detects binding events but not nucleotide-level contact points	TriFC/FRET is performed in cell; in vitro for purified components	Low	Low-moderate depending on fluorescent labeling	Section 2.1.5, Section 2.1.6 and Section 5.2.1
Immunoprecipitation-based methods (CLIP, RIP and related)	Allow identification of RNA targets of specific proteins (protein-centric) or protein partners of an RNA (RNA-centric). CLIP variants give nucleotide-level mapping after crosslinking and sequencing.	Require high-quality antibodies. CLIP requires UV crosslinking and extensive optimization.	High resolution for CLIP (precise crosslink sites). Low-medium for RIP (region-level)	CLIP is performed in cell; RIP is performed in cell/in vitro conditions	Moderate; depends on immunoprecipitation efficiency	Moderate; sequencing adds extra cost	Section 3.1
Crosslink-hybridization capture techniques (ChIRP, CHART, RAP)	RNA-centric capture with high specificity using antisense probes. Enables identification of protein partners of a given RNA and genomic binding sites.	Depends on probe design and crosslinking efficiency. Potential off-target hybridization.	High resolution for genomic/nucleic interaction maps (hybridization-guided)	Mainly in crosslinked cells	Moderate-high depending on probe sets	Moderate.	Section 3.2
Surface plasmon resonance (SPR)	Provides kinetic and thermodynamic parameters. Real-time, label-free detection.	Requires surface immobilization, which can bias interactions. Not always suitable for multi-component assemblies.	Medium resolution (binding affinity, not nucleotide-level positions)	In vitro conditions	Low-moderate	Moderate; requires SPR sensor system	Section 4
Enzyme/proximity labeling systems (APEX, BioID, TurboID)	Capture transient or weak interactions in living cells. Allow stringent purification due to covalent labeling. High spatial specificity if enzyme is correctly localized.	Require precise localization of the fusion protein. Risk of non-specific labeling if timing not optimized. Need high expression for efficient labeling.	Medium resolution (proximity window, not direct contact)	In live-cell labeling	Low-moderate	High; requires enzymes, substrates, and MS readout	Section 5.3

## Data Availability

No new data were created or analyzed in this study. Data sharing is not applicable to this article.
